# Level of partograph utilization and its associated factors among obstetric caregivers at public health facilities in East Gojam Zone, Northwest Ethiopia

**DOI:** 10.1371/journal.pone.0200479

**Published:** 2018-07-12

**Authors:** Desalegne Amare Zelellw, Teketo Kassaw Tegegne

**Affiliations:** 1 Department of Nursing, College of Medicine and Health Sciences, Bahir Dar University, Bahir Dar, Ethiopia; 2 Department of Public Health, College of Medicine and Health Sciences, Debre Markos University, Debre Markos, Ethiopia; TNO, NETHERLANDS

## Abstract

**Introduction:**

The discrepancy regarding maternal mortality continues to be a health concern between developing and developed countries. The majority of global maternal deaths occur in developing countries, specifically, in the sub-Sahara African region which alone accounts for more than half of these deaths. It has been indicated that utilization of the partograph was significantly associated with improved maternal and neonatal outcomes of labour and that is why the World Health Organization recommends the universal use of the tool during labour. Therefore, this study has assessed the level of partograph use and its associated factors among obstetric caregivers in East Gojam Zone, Northwest Ethiopia.

**Methods:**

A health facility based cross-sectional study was conducted among randomly selected obstetric caregivers in Northwest Ethiopia. The data were collected using a self-administered questionnaire and a clinical observation checklist. The data were entered into Epidata version 3.1, and cleaned and analyzed using SPSS version 24.0 statistical software.

**Result:**

About three quarters, or 198 (72.53%), of the obstetric caregivers, had attained diploma level of education. However, 153 (56.04%) of the obstetric caregivers had what was considered to be good knowledge about the partograph, but utilization of the tool was slightly lower than their level of knowledge, 147 (53.85%). Utilization of the partograph was significantly higher among obstetric caregivers holding a Bachelor of Science degree and above, than Diploma holders (AOR (95% C.I) 2.07 (1.15–3.75)) and the use was higher among those who were regularly working in the delivery ward compared to those regularly working in the Adult Outpatient Department (AOR (95% C.I): 2.25 (1.07–4.72)). Moreover, caregivers who had a good knowledge about the partograph and who had received on the job training in obstetric care were also more likely to use the partograph during labour and delivery (AOR (95% C.I): 1.79 (1.05–3.06) and 4.85 (2.63–8.96)) respectively.

**Conclusion:**

The results of this study revealed that although more than half of obstetric caregivers had a good knowledge of the partograph, the actual utilization of the tool was slightly lower than the knowledge they had. Therefore, in this study, we suggest that providing on the job obstetric care training for obstetric caregivers, about the partograph in particular, would improve partograph utilization.

## Background

The discrepancy between developing and developed countries in relation to maternal mortality continues to be reflected in the overall level of reproductive health care and its outcomes [1]. Globally, in 2015, the World Health Organization (WHO) estimated that there were 303,000 maternal deaths yielding a maternal mortality ratio (MMR) of 216 deaths per 100,000 live births. Here, about 99% (302,000) of the estimated global maternal deaths are from the developing regions, with the sub-Saharan Africa region alone accounting for about 66% (201,000) of those deaths [2]. The 2011 Ethiopian Demographic and Health Survey indicated that maternal mortality ratio was at 676 maternal deaths per 100,000 live births [3]. However, in 2015, the WHO estimated that the rate of mortality was expected to be reduced to 353 deaths per 100,000 live births [2].

Utilization of the partograph was related to improved maternal outcomes of labour [4]; hence, the World Health Organization recommends it to be used universally during labour [5]. Management of labour as early as possible has a good impact on maternal and foetal outcomes [6]. The partograph is an important tool in managing labour by generating a pictorial overview of the labour progress, and maternal and foetal condition, on a single sheet of paper, which allows the obstetric caregivers the opportunity to preemptively identify and diagnose symptoms of abnormal labour. Therefore, its use is critical in preventing and reducing the incidence of both maternal and perinatal morbidity and mortality by reducing unnecessary interventions and labour complication [1, 7].

In a systematic review carried out on causes of maternal mortality in Ethiopia, it was found that obstructed labour/uterine rupture, hemorrhage, hypertensive disorders of pregnancy and sepsis/infection, were the top four causes of maternal mortality [8]. The incidence of obstructed labour at Jimma University Specialized Hospital was 12.2%. The main causes of obstructed labour were Cephalo-Pelvic Disproportion (CPD) and Malpresentations accounted for 67.6% and 27.9% of cases respectively. The most common maternal complications were determined to be uterine rupture (45.1%) and sepsis (39.3%) [9].

In a study on the relationship between partograph use and birth outcome, researchers revealed that partograph use was associated with less maternal blood loss and neonatal injuries [10]. In an interventional study carried out on the impact of training on partograph use on maternal and perinatal outcome, it was found that partograph utilization was associated with a reduced rate of perinatal mortality, neonatal asphyxia, postpartum hemorrhage, obstructed labour, genital sepsis, and a better APGAR score at one and five minutes [11]. Moreover, in a case-control study conducted in India, it was found that the rate of emergency caesarean section was reduced from 44% in controls to 21% in cases, and none of the cases exhibited labour beyond 12 hours indicating a significant reduction of prolonged labour. A decrease in neonatal intensive care admissions (17% in controls to 6% in cases) was recorded, indicating an improvement in maternal and neonatal outcomes [4].

Even though the partograph has the above-mentioned importance, that is to make quick clinical decisions when problems arise in an expected normal birth, obstetric caregivers are not fully utilizing the tool [12]. In Ghana, about half of labouring mothers were not monitored using the partograph. On the other hand, only 40–60% of labouring mothers who were monitored with the partograph were monitored to standard, which implies a gap either in the skill in charting findings on the partograph up to the standard, or in appreciating the use of the tool in monitoring the progress of labour [13].

Similarly, in Ethiopia, there is no consistent use of the partograph during labour. In North Shoa, Central Ethiopia only 40.2% of the obstetric care providers routinely utilized the tool and its use was significantly associated with midwifery, having on the job training, knowledge and the attitude of the obstetric caregivers [14]. In another study carried out in Addis Ababa, Ethiopia it was shown that 57.3% of the obstetric care providers at the public health facilities had used the partograph to monitor mothers in labour. Here, utilization of the tool was significantly associated and higher among obstetric care providers working in health centres than those working in hospitals [15]. During labour, in the public health institutions of Bale Zone, Ethiopia, documentation of the modified WHO partograph was poor [16].

In Ethiopia, the major sources of maternal and neonatal morbidity and mortality are related to poor labour and delivery care. However, the partograph is used for early detection and prevention of maternal and foetal complications, but the level of utilization of the tool and the factors associated with it are not well studied in East Gojam Zone. Therefore, in this study, we aimed to determine the level of partograph use and identify the factors associated with its use among obstetric caregivers in East Gojam Zone, Northwest Ethiopia.

## Methods

### Study area

The study was conducted from March to July 2015 at 30 public health facilities (health centres and a hospital) in East Gojam Zone, Amhara Regional State, Northwest Ethiopia. Debre Markos is the capital city of East Gojam Zone, which is located at 299 Kilometers Northwest of Addis Ababa. In the zone, there are 19 districts, 101 health centres, and two hospitals. Concerning health workers, there are a total of 1417 health professionals with a qualification of midwifery, nurse, public health officer, medical doctor, and Master of Science degree (MSc) in emergency surgery and obstetrics.

### Study design

A health facility based cross-sectional study was conducted at 30 public health facilities in East Gojam Zone. The study was conducted among all health care professionals who were working in labour and delivery ward in a regular and/ or duty program at the selected public health facilities in East Gojam Zone.

### Sampling

A single population proportion formula was used to determine the sample size taking a 95% confidence interval, 5% margin of error, and a 26.6% proportion of the proper knowledge of the components of the partograph extracted from the previous study done in the Amhara Region [17]. This assumption gave a sample of 300, and hence the health workers in East Gojam zone were 1417, a reduction formula with a 10% non-response rate was used to get the final sample size of 273 obstetric caregivers.

Using the simple random sampling method, a total of 30 public health facilities (29 health centres and one hospital) were selected. The lists of all obstetric caregivers working in labour and delivery ward either on a regular and/or a duty program were used as a sampling frame. For each public health facility, a computer-generated simple random sampling technique with proportionate allocation to size was used to select the study participants.

In addition to the above-mentioned technique, 43 clinical observations (ranged from one to four obstetric caregivers per each sampled 30 health facilities) were carried out. All midwives, nurses, public health officers, medical doctors, and MSc in emergency surgery and obstetrics who were working in labour and delivery ward in a regular and/or a duty program were included in this study. However, health workers who were not at the health facility during the time of the visit were excluded from this study.

### Data collection

Both close-ended and open-ended structured self-administered questionnaires, prepared in English, were used. These were mainly focused on socio-demographic characteristics, profession and service year, types of health facilities, knowledge of the partograph, use of the partograph, current working department, and previous obstetric care training. On the other hand, for the clinical observations, a checklist adopted from the World Health Organization modified partograph [18] was used. There were ten specific items grouped under three broad categories, namely the progress of labour, foetal and maternal conditions. Under the progress of labour, there were three items: uterine contraction, descent and cervical dilation. The items under the foetal conditions were foetal heart rate, the moulding of the foetal skull and liquor. Finally, maternal pulse, maternal blood pressure, maternal temperature, and urine volume, ketone and protein were grouped under the maternal conditions (See S1 Questionnaire).

Health care providers who had the basic knowledge and skill of the partograph, and who worked in labour and delivery ward were recruited for data collection. A total of six data collectors: three nurses, two midwives, and one public health officer assisted the collection process. They were trained for two days about the objective of the study, the tool, the procedure of data collection and the rights of the study participants.

To maintain the quality of the data, the tool was adapted from previous studies and the modified WHO partograph, and training and a daily supportive supervision were given for the data collectors. Furthermore, before the actual conduction of the study, a pre-test was carried out and some changes were made to the tool. The completeness and consistency of the questionnaire were checked daily and appropriate corrections were taken accordingly.

Data collectors observed obstetric caregivers attending delivering mothers using the observation checklist. Efforts were made to minimize the effect of observation on providers behaviour, that is the Hawthorne effect [19], by assuring providers that the data collection was anonymous and individual performances would not be reported or shared publicly. Providers were not aware of what topics and items were on the checklist, so they could not prepare in any way. Observers did not visit health facilities where they are currently working or previously worked as clinicians, to minimize the effect of personal and professional relationships.

### Data analysis

The collected data were entered using Epidata version 3.1. Data cleaning and analysis were carried out using SPSS version 24.0 software. Descriptive statistics were carried out using frequencies, percentages, mean, median and standard deviation. As described elsewhere [20], obstetric caregivers knowledge of the partograph was assessed based on eight knowledge specific questions; 0–4 and 5–8 points were categorized as having a poor and a good knowledge, respectively. A binary logistic regression with a 95% confidence interval was run to assess any significant association between the dependent (use of the partograph) and independent variables (socio-demographic characteristics, profession and service year, types of health facilities, knowledge of the partograph, current working department, and previous obstetric care training). To control the potential effects of confounders and not to miss important variables, independent variables with a p-value of less than 0.20 at the bivariate analysis were entered into the multivariable logistic regression model. In this model, the level of significance was determined at a 95% confidence interval with a p-value of less than 0.05. The fitness of the regression model was tested using the Hosmer-Lemeshow goodness of fit.

### Ethical consideration

The ethical paper was obtained from the Debre Markos University Ethical Review Committee. A formal letter of permission and support was obtained from East Gojam Zone Health Department and District Health Offices. Permission to conduct the study was obtained from the respective health facilities. Data were collected after obtaining an informed written consent from each obstetric caregiver. Moreover, for the clinical observations, informed written consent was obtained from both the obstetric caregiver and the delivering mother. The study participants were given the full right to withdraw from the study at any time without any form of preconception, and their confidentiality was maintained.

## Results

### Socio-demographic characteristics of obstetric caregivers

Two hundred and seventy-three obstetric caregivers participated in this study. More than half of the study participants were males. The mean age of the study participants was 27.64 (with a standard deviation of ±4.50) years. About one-quarter of the caregivers were below 25 years. One hundred and fifty-eight (57.88%) of the participants were single. With regard to educational status, nearly three quarters (72.53%) of the obstetric caregivers attained a diploma level of education (Table 1).

**Table 1:** Socio demographic characteristics of obstetric caregivers in East Gojam Zone, Northwest Ethiopia, 2015 (n = 273).

Variables		Frequency	Percentage
**Sex**	Male	157	57.51
	Female	116	42.49
**Age Group**	< = 24 Years	71	26.01
	25–29 Years	128	46.89
	> = 30 Years	74	27.11
**Marital Status**	Single	158	57.88
	Married	115	42.12
**Educational Level**	Diploma	198	72.53
	BSc Degree and above	75	27.47

### Obstetrics caregivers healthcare characteristics

The majority (246 or 90.1%) of obstetrics caregivers were working in health centres and more than one fifth, 60 (22.0%) were Diploma Midwives. One hundred and forty-one (51.6%) of the caregivers were working in the delivery ward regularly while the rest were working during night duty and/or at the weekend. Nearly half (132 or 48.4%) of obstetric caregivers had a maximum of three years of clinical service and only 91 (33.3%) had on the job training in obstetric care. Among those who had on the job training in obstetric care, only 57 (62.6%) had received training on Basic Emergency Obstetric Care (BEMOC). On the other hand, only 78 (28.6%) had received in-service or refresher training on the partograph directly or indirectly (Table 2).

**Table 2:** Obstetric caregivers healthcare characteristics in East Gojam Zone, Northwest Ethiopia, 2015 (n = 273).

Variables		Frequency	Percentage
**Profession**	Diploma Nurse	138	50.5
	Diploma Midwife	60	22.0
	BSc Public Health	37	13.6
	BSc Nurse	17	6.2
	BSc Midwife	14	5.1
	Others^†^	7	2.6
**Regular Working Department**	Delivery Ward	141	51.6
	Antenatal Care	47	17.2
	Family Planning	36	13.2
	OPD (Adult &/ Under-five)	49	17.9
**Years of Clinical Service**	< = 3 Years	132	48.4
	4–6 Years	87	31.9
	> = 7 Years	54	19.8
**Training on Obstetric Care**^††^	Yes	91	33.3
	No	182	66.7
**Type of Training**	Basic Emergency Obstetric Care *(BEMOC)*	57	62.6
	Newborn Care	34	37.4
**Studied Partograph**^†††^	Yes	192	70.3
	No	81	29.7
**Training on Partograph**	Yes	78	28.6
	No	195	71.4

^†^Others (medical doctors and MSc in emergency surgery and obstetrics)

^††^On the job training

^†††^preservice training

### Utilization of partograph

Even though 153 (56.04%) of the caregivers had a good knowledge about the partograph, only 147 (53.85%) had used the tool. Out of the caregivers who used the partograph, 54 (36.7%), 52 (35.4%) and 41 (27.9%) of them had used it routinely, sometimes and occasionally, respectively. Although more than half of caregivers had used the partograph, there was variation in plotting all the components of the partograph. Only 85 (57.8%) obstetric caregivers had plotted the foetal heart rate every 30 minutes, and more surprisingly, only 35 (23.8%) of them had plotted correctly across the action line (Table 3).

**Table 3:** Utilization of partograph by obstetric caregivers at public health facilities in East Gojam Zone, Northwest Ethiopia, 2015 (n = 147).

Variables	Frequency	Percentage
Plot foetal heart rate every 30 minutes	85	57.8
Plot initial cervical dilation	74	50.3
Plot cervical dilation every 4 hours	65	44.2
Plot descent	61	41.5
Plot uterine contraction	60	40.8
Record membrane intact or ruptured	60	40.8
Record color of liquor	58	39.5
Monitor maternal blood pressure every 4 hours	59	40.1
Monitor maternal pulse every 30 minutes	46	31.3
Plot correctly across alert line	42	28.6
Plot correctly across action line	35	23.8

**Fig 1:**
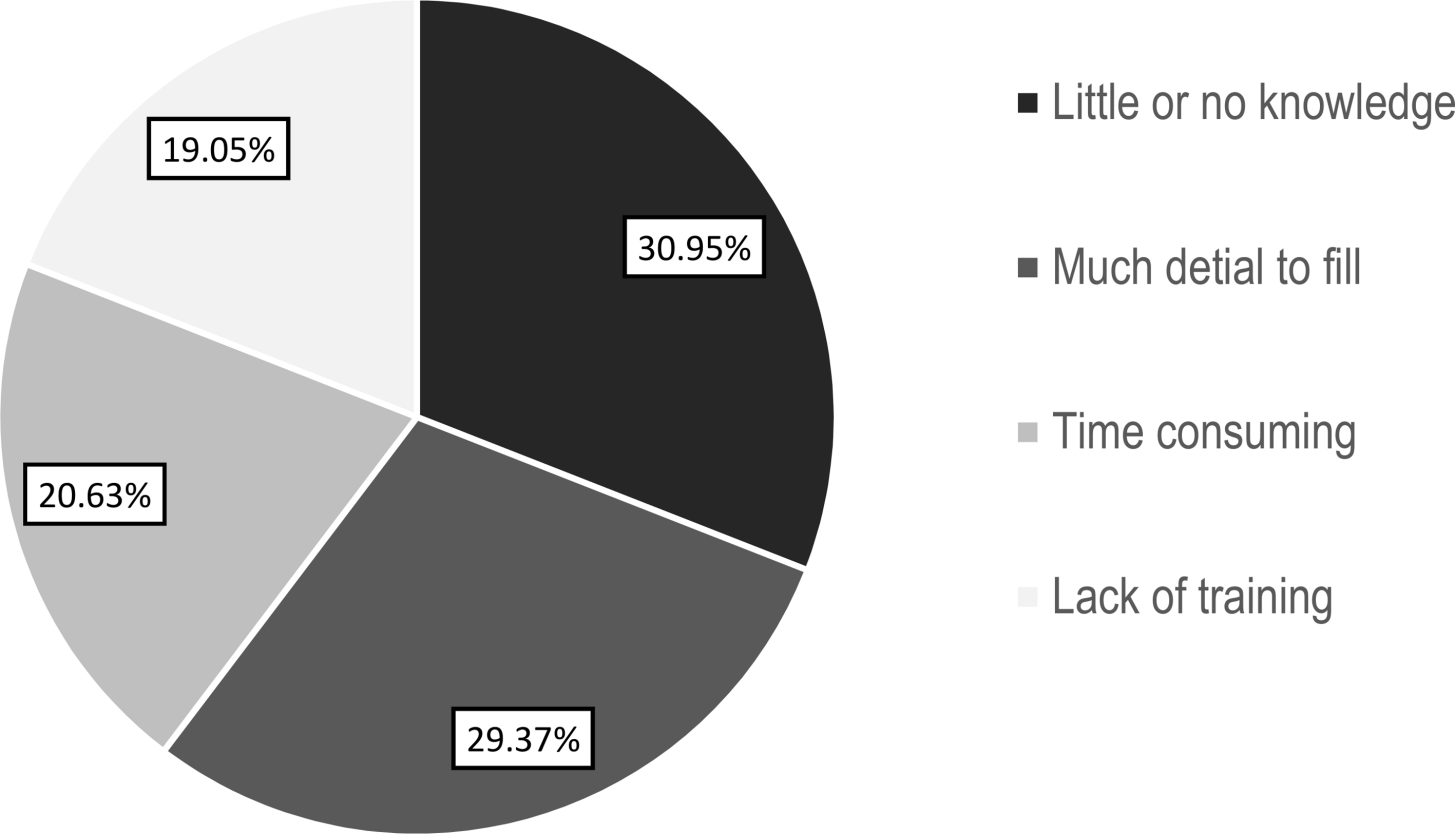
Reasons for obstetric caregivers for not using partograph at public health facilities in East Gojam Zone, Northwest Ethiopia, 2015 (n = 126).

One hundred and twenty-six (46.15%) of the obstetric caregivers did not use the partograph. Amongst these caregivers, 39 (30.95%) had mentioned that lack of knowledge was their main reason for not using the partograph (Fig 1).

### Observation of partograph use

Most (39) of the clinical observations were conducted at the health centres while only four of them were at the hospital. The majority of the observed births were attended by female nurses and midwives. It was found that none of the components of the partograph was completely used to monitor the labouring mother. The maternal condition was the most ignored component compared to the other two components of the partograph. Surprisingly, out of the 43 observations, only one delivering mother’s urine was checked for its volume, protein and ketone bodies (Table 4).

**Table 4:** Observation of partograph utilization at public health facilities in East Gojam Zone, Northwest Ethiopia, 2015.

Parameters	Yes
**Progress of labour**	
Check and plot cervical dilation every 4 hours	30
Check and plot descent of head	27
Check and plot uterine contraction every ten minutes	25
**Foetal condition**	
Monitor and plot foetal heart rate every 30 minutes	28
Check and record colour of liquor during every per vaginal examination	30
Check and plot moulding of foetal skull	19
**Maternal condition**	
Monitor and plot maternal pulse rate every 30 minutes	10
Monitor and plot maternal blood pressure every 4 hours	13
Monitor and plot maternal temperature every 2 hours	6
Monitor and record urine volume, urine protein and ketone every 2–4 hours	1

### Factors associated with utilization of partograph

According to the multivariable analysis, obstetric care providers holding a Bachelor of Science degree (BSc degree) and above were 2.07 times more likely to use the partograph than diploma holders (AOR (95% C.I) 2.07 (1.15–3.75)). Obstetric caregivers who were working in the delivery ward regularly were 2.25 times more likely to use the photograph than those regularly working in an adult outpatient department (AOR (95% C.I): 2.25 (1.07–4.72)). Moreover, caregivers who had good knowledge about the partograph were 1.79 times more likely to use partograph during labour and delivery than those who had poor knowledge about the partograph (AOR (95% C.I): 1.79 (1.05–3.06)). Similarly, obstetric caregivers who had received on the job training were also 4.85 times more likely to use the partograph than their counterparts (AOR (95% C.I): 4.85 (2.63–8.96)) (Table 5). However, the variation in professions and acquisition of knowledge about the partograph at college or university level were confounding factors on partograph use.

**Table 5:** Factors associated with utilization of partograph, East Gojam Zone, Northwest Ethiopia, 2015(n = 273).

Variables		Partograph Use		Crude Odds Ratio (95% C.I.)	Adjusted Odds Ratio (95% C.I.)
		No	Yes		
		n (%)	n (%)		
**Sex**	Female	59 (50.86)	57 (49.14)	1.00	1.00
	Male	67 (42.68)	90 (57.32)	1.39 (0.86–2.25)	1.23 (0.71–2.12)
**Educational Level**	Diploma	97 (48.99)	101 (51.01)	1.00	1.00
	BSc Degree and above	29 (38.67)	46 (61.33)	1.52 (0.89–2.62)	2.07 (1.15–3.75)*
**Profession**	Nurse	88 (55.70)	70 (44.30)	1.00	1.00
	Midwife	25 (32.05)	53 (67.95)	2.67 (1.51–4.71)**	1.21 (0.59–2.48)
	BSc Public Health	13 (35.14)	24 (64.86)	2.32 (1.10–4.89)*	1.66 (0.60–4.59)
**Regular Working Department**	OPD (Adult &/Under-Five)	30 (61.22)	19 (38.78)	1.00	1.00
	Delivery Ward	54 (38.30)	87 (61.70)	2.54 (1.31–4.96)**	2.25 (1.07–4.72)*
	Antenatal Care	20 (42.55)	27 (57.45)	2.13 (0.94–4.82)	2.29 (0.92–5.69)
	Family Planning	22 (61.11)	14 (38.89)	1.01 (0.42–2.43)	1.25 (0.48–3.30)
**Obstetric Training**^††^	No	105 (57.69)	77 (42.31)	1.00	1.00
	Yes	21 (23.08)	70 (76.92)	4.55 (2.57–8.03)***	4.85 (2.63–8.96)***
**Studied Partograph**^†††^	No	53 (65.43)	28 (34.57)	1.00	1.00
	Yes	73 (38.02)	119 (61.98)	3.09 (1.79–5.31)***	1.64 (0.88–3.05)
**Knowledge about Partograph**	Poor Knowledge	68 (56.67)	52 (43.33)	1.00	1.00
	Good Knowledge	58 (37.91)	95 (62.09)	2.14 (1.32–3.49)**	1.79 (1.05–3.06)*

Significant at

*p-value < 0.05

**p-value < 0.01

***p-value < 0.001.

^††^On the job training

^†††^preservice training

## Discussion

In this study, the level of partograph utilization was slightly lower than the obstetric caregivers grouped knowledge score. This finding was similar to the study done in Addis Ababa, Ethiopia [15], but it was lower than other studies carried out in North Shoa [14], Benin [21], the Niger Delta of Nigeria [22], South Africa [23], and Gambia [24]. However, it was higher than other studies done in Ethiopia [25, 26] and Nigeria [1, 27, 28]. These differences might be attributed to differences in the study area, as there might be different policy, strategy and commitment towards using the tool routinely for every labouring mother; even this might differ at the various levels within a country. Furthermore, time by itself has its own contribution, the functioning policy and implementation strategy might change over time. In addition, the difference in study participants might bring a difference in using the partograph. Health care providers’ low awareness of the partograph [28, 29], and the knowledge and skill deficit in maternity care [12, 28, 30–32] were barriers in the use of the partograph. A study carried out in Addis Ababa, Ethiopia found that lack of partograph knowledge, negative attitudes like it is much detail to fill and the assumption that it is time-consuming and medical doctors’ work, understaffing, and lack of training were the barriers in the use of the tool [15].

Even though more than half of the obstetric caregivers used the partograph, its utilization was not consistent everywhere and every time. The tool was not routinely used; and even among those who used the partograph, it was not used according to the recommended standard. This finding was supported by different studies carried out in Benin [21], Dare es Selaam [33] and Uganda [31]. These could be related to the knowledge gap, skill incompetency, workload or shortage of staff, lack of motivation, negligence, and a shortage of resources and infrastructure. For instance, a health facility with inadequate or dysfunctional medical equipment, low number of beds, inadequate rooms, and lack of laboratory reagents and kits might be related to the inappropriate use of the partograph or even may not be used at all. Due to the increased flow of patients coupled with a shortage of staff and inadequate rooms and beds, it would be difficult to use the tool according to the standard. In a systematic review carried out on the partograph, it was found that obstetric care providers’ use of the partograph was related to the innovations or changes that are made on the tool, the caregivers difference in knowledge, attitude, awareness and confidence to use the tool, the regular clinical supportive supervision and quality assurance, and each and every organizations context of obstetric care [34].

The utilization of the partograph was significantly higher among obstetric caregivers holding a Bachelor of Science (BSc) degree and above, compared to diploma holders. It might be due to the fact that BSc degree and above holders might have good comprehensive knowledge of the partograph and/or they might be midwives too. In health centres, they might also have a better chance of being consulted on obstetric conditions, and having the knowledge and skill required to use the partograph in identifying abnormal labour progress early, as well as arranging for the timely referral to higher health facilities, might be their responsibility too. Moreover, they might have refresher training on the partograph or obstetric care as demonstrated by the positive relationship as it pertains to the use of the partograph [35].

Furthermore, partograph utilization was significantly higher among obstetric care providers who had ever received on the job training in obstetric care. This finding was supported by another study carried out in North Shao, Ethiopia [14] and Nigeria [11, 35]. It is obvious that obstetric caregivers who had received on the job training on the partograph might have a better understating about the tool and thus more likely to use it during labour. It was observed that on the job training was significantly associated with a good knowledge of the partograph [36], and in turn being knowledgeable of the partograph was significantly associated with utilization of the tool during labour [14]. However, use of different monitoring tools, unavailability of the partograph, and a shortage of staff and lack of trained caregivers were the reasons for not using the tool during labour [14].

Utilization of the partograph was marginally significant among obstetric caregivers who had good knowledge about it. This was supported by another study as knowledge of the partograph had a statistically significant association in utilizing the tool [14, 35]. Having good knowledge about the partograph might enhance obstetric caregivers’ skills and competency to use the tool properly. However, health care providers’ attitude and limited confidence [31, 37], variation in their commitment [38] and poor interaction with delivering mother [39] were related to underutilization of the tool. Furthermore, lack of available guidelines [31], understaffing and high workload [31, 32, 40], frequent staff rotation [39] and job dissatisfaction [41] were the other barriers for the underutilization of the partograph.

Even though it was marginally significant, utilization of the partograph was higher among obstetric caregivers who were working in the delivery ward routinely. This might be due to the fact that obstetric caregivers assigned in delivery wards could be midwives and/or degree holders who might have a better chance to receive on the job training in obstetric care. In turn, they could acquire and improve their knowledge and skills of the partograph. This was supported by a study carried out in North Shoa, Central Ethiopia, where being a midwife by profession was significantly associated with utilization of the partograph during labour [14].

## Limitations

Due to the cross-sectional nature of the study, it doesn’t establish a causal association between the partograph utilization and the independent variables. The data were collected using a self-administered questionnaire and it could be subjected to social desirability bias. Moreover, even though efforts were made to minimize observation bias, the possibility of Hawthorne effect is likely. In addition to this, use of the proportionate allocation has its own limitation; the chance of the study participants being included in the sample is not the same for everyone. The smaller the size of the sample, the lower the chance of the study participants to be included in the study and vis-versa.

## Conclusion

The results of this study revealed that although more than half of obstetric caregivers had a good knowledge of the partograph, the actual utilization of the tool was slightly lower than the knowledge they had. Partograph utilization was higher among obstetric caregivers holding a BSc degree and above, had in-service obstetric care training, had a good knowledge about the partograph, and who were regularly working in the labour and delivery ward. Therefore, in this study, we suggest that providing on the job obstetric care training for obstetric caregivers, about the partograph in particular, would improve partograph utilization. Furthermore, regular supportive supervision will be important to improve obstetric care in general, and proper utilization of the partograph and to help obstetric caregivers become dedicated to record and document their findings in particular.

## Supporting information

S1 Questionnaire Questionnaire on partograph knowledge and utilization. (DOCX)
