# Supplementary material for: Level of partograph utilization and its associated factors among obstetric caregivers at public health facilities in East Gojam Zone, Northwest Ethiopia
Source: PLoS One. 2018 Jul 12;13(7):e0200479. doi: 10.1371/journal.pone.0200479 (PMC6042737; doi:10.1371/journal.pone.0200479)
Supplement: S1 Questionnaire — (DOCX) [file pone.0200479.s001.docx]

**S1 Questionnaire: Questionnaire on partograph knowledge and utilization**

**Section I: Self-administered questionnaire**

***General instruction:*** *We kindly request you to circle or write your response on the space provided for each questions accordingly. Please, keep in mind that there are some skipping patterns that you will follow too.*

**Part 100: Socio-demographic characteristics**

| **Ques Code** | **Question (variable)** | **Response** | **Skip** | **Response code** |
| --- | --- | --- | --- | --- |
| 101 | Sex | 1. Male 2. Female |  |  |
| 102 | Age (in full years) | ---------------------- |  |  |
| 103 | Religion | 1. Orthodox Christians 2. Muslim 3. Protestant 4. Catholic   88. Others (specify) ------------------ |  |  |
| 104 | Marital Status | 1. Single (Never married) 2. Married 3. Widowed 4. Separated 5. Divorced |  |  |
| 105 | Educational Level | 1. Diploma 2. Degree 3. MSc in Emergency Surgery and Obstetrics 4. Specialist (Obstetrician and Gynecologist) |  |  |

# Part 200: Obstetric caregivers’ health care characteristics

| **Ques Code** | **Question (variable)** | **Response** | **Skip** | **Response code** |
| --- | --- | --- | --- | --- |
| 201 | Profession | 1. Diploma Nurse 2. BSc Nurse 3. Diploma Midwife 4. BSc Midwife 5. Public Health (HO) 6. Medical Doctor (GP) 7. Emergency Surgery and Obstetrician (MSc) 8. Obstetrician and Gynecologist (Specialist) |  |  |
| 202 | Health Facility | 1. Health Center 2. Hospital |  |  |
| 203 | Regular working department | 1. Delivery room 2. ANC 3. PNC 4. Family Planning 5. OPD (Adult &/ Under-five)   88. Others (Specify) ------------------- |  |  |
| 204 | Ever worked in labor and delivery room? | 1. Yes 2. No |  |  |
| 205 | Total years of clinical service | _____________ (full year) |  |  |
| 206 | Ever received training on obstetric Care? | 1. Yes 2. No ------------------------------------------ | 208 |  |
| 207 | Which training did you received? | 1. Labor 2. Delivery 3. Newborn care 4. BEMONC 5. CEMONC   88. Others |  |  |
| 208 | Did you study partograph? (as part of academic education) | 1. Yes 2. No |  |  |
| 209 | Did you trained to use partograph? (in service training) | 1. Yes 2. No |  |  |

# Part 300: knowledge about partograph

| **Ques Code** | **Question (variable)** | **Response (list all the responses using serial numbers)** | **Response code** |
| --- | --- | --- | --- |
| 301 | What is partograph? | ------------------------------------------------------------------------------------------------------------------------------------------------------------------------------------------------------------------------------------------------------------------------------------------------------------------------------------------------------------------------------------------------------------------------------------------- |  |
| 302 | What are the components of the Partograph? | 1. -------------------------------------------------------------------------- 2. -------------------------------------------------------------------------- 3. -------------------------------------------------------------------------- 4. -------------------------------------------------------------------------- 5. -------------------------------------------------------------------------- 6. -------------------------------------------------------------------------- 7. -------------------------------------------------------------------------- 8. -------------------------------------------------------------------------- 9. -------------------------------------------------------------------------- 10. -------------------------------------------------------------------------- 11. -------------------------------------------------------------------------- |  |
| 303 | Why do you use the partograph or what is its use? | 1. -------------------------------------------------------------------------- 2. -------------------------------------------------------------------------- 3. -------------------------------------------------------------------------- 4. -------------------------------------------------------------------------- 5. -------------------------------------------------------------------------- |  |
| 304 | What is the function of Alert Line? | 1. -------------------------------------------------------------------------- 2. -------------------------------------------------------------------------- 3. -------------------------------------------------------------------------- 4. -------------------------------------------------------------------------- 5. -------------------------------------------------------------------------- |  |
| 305 | What is the functions of Action Line? | 1. -------------------------------------------------------------------------- 2. -------------------------------------------------------------------------- 3. -------------------------------------------------------------------------- 4. -------------------------------------------------------------------------- 5. -------------------------------------------------------------------------- |  |
| 306 | When do be plotting on the Partograph started? | 1. -------------------------------------------------------------------------- 2. -------------------------------------------------------------------------- 3. -------------------------------------------------------------------------- 4. -------------------------------------------------------------------------- 5. -------------------------------------------------------------------------- |  |
| 307 | What do mean by satisfactory labour progress? | 1. -------------------------------------------------------------------------- 2. -------------------------------------------------------------------------- 3. -------------------------------------------------------------------------- 4. -------------------------------------------------------------------------- 5. -------------------------------------------------------------------------- |  |
| 308 | What is the importance of Partograph | 1. -------------------------------------------------------------------------- 2. -------------------------------------------------------------------------- 3. -------------------------------------------------------------------------- 4. -------------------------------------------------------------------------- 5. -------------------------------------------------------------------------- |  |

**Part 400: Assessment of utilization of the partograph**

| **Ques Code** | **Question (Components of the partograph plotted)** | **Response** | **Skip** | **Response code** |
| --- | --- | --- | --- | --- |
| 401 | Do you use partograph? | 1. Yes 2. No -------------------------------- | 414 |  |
| 402 | How often do you use? | 1. Routinely 2. Some times 3. Occasionally |  |  |
| 403 | Did you plot fetal heart rate correctly? | 1. Yes 2. No |  |  |
| 404 | Did you plot cervix initial dilation correctly | 1. Yes 2. No |  |  |
| 405 | Did you plot cervical dilation 4 hourly? | 1. Yes 2. No |  |  |
| 406 | Did you plot descent correctly? | 1. Yes 2. No |  |  |
| 407 | Did you plot uterine contraction correctly? | 1. Yes 2. No |  |  |
| 408 | Did you record membrane intact or ruptured? | 1. Yes 2. No |  |  |
| 409 | Did you record color of liquor correctly? | 1. Yes 2. No |  |  |
| 410 | Did you monitor maternal B/P at least 4 hourly? | 1. Yes 2. No |  |  |
| 411 | Did you monitor maternal pulse at least every 30 minutes? | 1. Yes 2. No |  |  |
| 412 | Did you plot correctly across alert line? | 1. Yes 2. No |  |  |
| 413 | Did you plot correctly across action line? | 1. Yes 2. No |  |  |
| 414 | If not using, what are the reasons? | 1. Little or no knowledge 2. Much detail to fill 3. Time consuming 4. Shortage of staff 5. Doctor’s do that 6. Lack of training 7. None availability   88. Others |  |  |

**Section II: Observation checklist**

*Please record whether the provider performed any of the following procedures while attending labour and delivery (circle* ***YES****, or* ***NO****). Write any additional comments you observe.*

| **Parameters** | **Response** | |
| --- | --- | --- |
| **Progress of labour** | | |
| Check and plot cervical dilation every 4 hour | **Yes** | **No** |
| Check and plot descent of head | **Yes** | **No** |
| Check and plot uterine contraction every ten minute | **Yes** | **No** |
| **Foetal condition** | | |
| Monitor and plot foetal heart rate every 30 minutes | **Yes** | **No** |
| Check and record colour of liquor during every per vaginal examination | **Yes** | **No** |
| Check and plot moulding of foetal skull | **Yes** | **No** |
| **Maternal condition** | | |
| Monitor and plot maternal pulse rate every 30 minutes | **Yes** | **No** |
| Monitor and plot maternal blood pressure every 4 hour | **Yes** | **No** |
| Monitor and plot maternal temperature every 2 hours | **Yes** | **No** |
| Monitor and record urine volume, urine protein and ketone every 2-4 hours | **Yes** | **No** |

***Comment:*** ------------------------------------------------------------------------------------------------------------------------------------------------------------------------------------------------------------------------------------------------------------------------------------------------------------------------------------------------------------------------------------------------------------------------------------------------------------------------------------------------------------------------------------------------------------------------------------------------------------------------------------------------------------------------------------------------------------------------------------------------------------------------------------------------------------------------------------------------------------------------------------------------------------------------------------------------------------------------------------------------------------------------------------------------------------------------------------------------------------------------------------------------------------------------------------------------------------------------------------------------------------------------------------------------------------------------------------------------------------------------------------------------------------------------------------------------------------------------------------------------------------------------------------------------------------------------------------------------------------------------------------------------------------------------------------------------------------------------------------------------------------------------------------------------------------------------------------------------------------------------------------------------------------------------------------------------------------------------------------------------------------------------------------------------------------
